# Exploring the interplay among smoking, stress, and negative affect in patients with psychosis: An experience sampling method study

**DOI:** 10.1192/j.eurpsy.2025.10148

**Published:** 2025-12-29

**Authors:** Dunja van der Velde, Sanne van der Heijden, Claudia Simons, Therese van Amelsvoort, Marieke van der Pluijm, Behrooz Alizadeh, Wim Veling, Lieuwe de Haan, Wiepke Cahn, Frederike Schirmbeck, Jentien Vermeulen

**Affiliations:** 1 Amsterdam UMC location AMC, Netherlands; 2 Maastricht University Medical Center, Netherlands; 3 University Medical Center Groningen, Netherlands; 4 University Medical Center Utrecht, Netherlands; 5 University Hospital Mannheim of University of Heidelberg Faculty of Medicine, Germany

**Keywords:** experience sampling method, negative affect, psychosis, smoking, stress

## Abstract

**Background:**

Tobacco smoking is highly prevalent in patients with psychosis, who also often experience negative affect (NA) and stress. The relationship between these factors remains unclear in this population. We aimed to investigate everyday life associations in 158 patients with psychosis, 136 unaffected siblings, and 117 controls from the Genetic Risk and Outcome of Psychosis (GROUP) study with Experience Sampling Method measurements.

**Methods:**

Generalized linear mixed models were used to evaluate across time and within-subject associations. Across time analyses investigated the relationship between smoking status and overall NA and stress. Within-subject analyses assessed whether smoking between two measurements (*t*_−1_ and *t*_0_) was associated with changes in NA and stress at the measurement after smoking a cigarette (*t*_0_) and at the subsequent measurement (*t*_+1_).

**Results:**

Across assessments, smoking status was initially associated with NA in patients (*B*=0.26, *p*=0.036), but this association disappeared after controlling for psychotic symptoms and cannabis use. Within-subject analyses in smokers showed a decrease in NA in patients after smoking (*t*_0_: −0.23, *p*=0.016), which remained significant after correcting for confounders (*t*_0_: −0.20, *p*=0.015). Siblings showed a decrease in NA (*t*_0_: −0.22, *p*=0.009), also after controlling for confounders (*t*_0_: −0.14, *p*=0.018). No time-lagged effect was found at *t*_+1_ after correction for subsequent smoking.

**Conclusions:**

Overall smoking behavior was not associated with NA in patients with psychosis. In the short term, smoking in the daily life context is associated with a reduction in NA in people vulnerable to psychosis, possibly due to alleviation of withdrawal symptoms, which may complicate smoking cessation.

## Introduction

Patients with psychosis are more vulnerable to stress, even in situations considered non-stressful [1]. They also report higher negative affect (NA, i.e. the experience of subjective distress in everyday life) than healthy controls [2]. In patients with psychosis, cross-sectional multilevel models showed that an increase in stress was associated with higher NA, which was then associated with more intense psychotic symptoms, such as paranoia, hallucinations, and loss of control [3]. Smoking, a modifiable lifestyle factor, is linked to both stress and NA [4], and its prevalence is three times higher in patients with psychosis than in the general population [5]. This suggests a potential link between smoking, stress, NA, and psychosis.

Patients with psychosis subjectively report that they smoke cigarettes to alleviate stress and NA [6], consistent with the self-medication hypothesis [7]. However, literature on the self-medication hypothesis in patients with psychosis has been refuted. Amelioration of symptoms like irritability, anxiety, and depression after smoking a cigarette may be misattributed to mental illness, which are actually withdrawal symptoms of nicotine dependence [8]. Based on this misattribution, patients may see smoking as a coping strategy to deal with affective symptoms [9, 10]. This may explain why patients (and clinicians) have long feared that smoking cessation might worsen psychiatric symptoms and why smoking cessation is more difficult in this group. In this line, prior research frequently referred to NA as a barrier to smoking cessation [9, 11]. The difficulty of quitting is likely due to higher nicotine dependence, more intense withdrawal symptoms [9], and smoking as a coping strategy [10]. On the contrary, longitudinal cohort studies and systematic reviews suggest that patients with psychosis who quit smoking do not show worsening of symptom severity, but improvement on the overall level of symptoms, stress, cognitive functioning, and quality of life [12–15]. These findings suggest that perceived subjective benefits of smoking may reflect the temporary relief of withdrawal symptoms. Despite these findings, it remains unclear whether smoking alleviates or aggravates stress and NA in daily life. One large prospective study in middle-aged smokers in the general population found that they consumed, on average, more cigarettes on stressful days compared to non-stressful days, and experienced higher levels of NA on days when they smoked more than usual [16]. These findings are in line with the stress-induction model of smoking [17], which proposes that smoking leads to an increase in stress and NA. However, it is also possible that stress or NA leads to smoking.

Previous literature on patients with psychosis has focused on overall smoking prevalence [5], motives to smoke [6], or the effects of cessation [14]. However, to the best of our knowledge, no study has examined the associations between smoking status and stress or NA, or how smoking and momentary experiences of stress and NA influence each other in daily life in psychosis. Hence, further exploration is needed into the exact nature and direction of the relationship between smoking, stress, and NA. Understanding this interplay is crucial, since stress and NA play a role in relapse and symptom exacerbation in psychosis [18], and smoking is a potentially modifiable lifestyle factor. Such knowledge could inform smoking cessation interventions tailored to patients with psychosis, for instance, by addressing stress and NA as barriers to quitting and by reframing smoking as a factor that maintains, rather than alleviates, stress.

The experience sampling method (ESM) provides the possibility to investigate the temporal sequence between smoking at a specific time point and a change in stress and NA afterwards [18–20]. Within the Genetic Risk and Outcome of Psychosis (GROUP) study, patients, unaffected siblings, and healthy controls reported ESM data on six consecutive days. These data make it possible to explore these time-lagged associations in individuals with different levels of psychosis liability [21]. Based on the previously mentioned literature, we aimed to test the hypotheses that:

1. Across ESM assessments, smokers show higher levels of NA and experience stress in everyday life compared to those who do not smoke.
2. Among participants who smoke, the event of smoking is associated with an increase in NA and experienced stress at the following assessment.

## Method

### Study design

The GROUP study is a six-year longitudinal cohort study across four Dutch academic centers and affiliated mental health services in the Netherlands and (the Dutch-speaking part of) Belgium [21]. Patients were included if they were diagnosed with a non-affective psychotic disorder according to the Diagnostic and Statistical Manual of Mental Disorders, Fourth Edition (DSM-IV) criteria [22]. Siblings and healthy controls were included if non-affected by a lifetime psychotic disorder. Other inclusion criteria were age at inclusion between 16 and 50 years old (extremes included), good command of the Dutch language, and being able and willing to give written informed consent. The study protocol was approved by the Ethical Review Board of the University Medical Centre Utrecht. For this study we used ESM measures as part of the data collection within the GROUP study, which were performed at six year follow-up (T3) in a subset of participants from the whole GROUP sample.

### Data collection

#### Sociodemographic and clinical characteristics

For sample characteristics, assessments of age, sex, ethnicity, level of education and marital status were carried out at the moment of the ESM measurements, at T3 in the GROUP study. The Community Assessment of Psychic Experience (CAPE), frequency measures [23] were assessed as a self-report of psychotic experiences within the past three years. Each of the items was rated on a scale from 0 (absent) to 3 (almost always present). A mean total score was calculated for the subscales of positive symptoms (if at least 14 of 20 items were available), negative symptoms (if at least nine of 14 items were available), and depressive symptoms (if at least five of eight items were available). For patients, the Positive and Negative Symptoms Scale (PANSS) was measured for symptom severity [24]. Three of five available dimensions were used: positive symptoms (range 1–55), negative symptoms (range 2–62), and emotional distress (8–56).

#### ESM measures

ESM is a structured diary method with high ecological validity that provides moment-to-moment information prospectively [25]. Therefore, a technological device (the PsyMate; www.psymate.eu [26]) was used to assess emotions, events, and symptoms within the real-time everyday life context. At random times between 7:30 and 22:30, prompts occurred ten times per day (approximately 90 minutes apart) over six consecutive days. Participants were instructed to complete the ESM assessment right after the prompt, within 15 minutes, to minimize memory distortions. Participants were asked to fill in several questionnaires about, for example, emotional states, activities, and substance use. Based on previous research using ESM in patients with schizophrenia, participants were included if they provided ESM data with at least 20 valid responses across six consecutive days [27]. In accordance with previous literature [3], the following outcome variables were defined, with NA and the stress assessed through ESM items rated on a 7-point Likert scale, where higher scores indicate higher levels of NA and stress (for some variables, recoding and reverse scoring were necessary).

##### Substance use

At each prompt, participants reported cigarette and cannabis use since the previous prompt (yes/no). For across-time analyses, smoking status was derived from the ESM measures. Someone was defined as a smoker if they smoked at least one measurement during the six-day assessment period. Thus, not all participants were classified as smokers; both smokers and non-smokers were included in across-time analyses, while only smokers were included in the within-subject analyses. For descriptives, we derived an index of cigarettes per day from the number of prompts at which smoking was reported. Data on smoking history were not available.

##### Negative affect

The variable NA was defined as the mean of the following five ESM items: “I feel… insecure, down, lonely, anxious, and irritated” [3]. Cronbach’s alpha for NA was 0.93.

##### Event stress

Event-related stress was based on one ESM item asking participants to rate how pleasant their most important event since the last measurement was [3].

##### Activity stress

For activity-related stress, the mean of three ESM items was included: “I would prefer to do something else”, “This activity is difficult for me”, and “I can do this well” [3]. Cronbach’s alpha of the variable activity stress was 0.76.

##### Social stress

The rating of social stress was scored depending on whether a participant was in the company of others or alone [3]. The first ESM question assessed the context by asking “Who am I with?” (alone, partner, residents, family living elsewhere, friends, colleagues, health care professional, acquaintances, or strangers/others). If alone: “I would prefer company” and “It is pleasant to be alone.” If with company: “I would prefer to be alone” and “I find these people pleasant.” Cronbach’s alpha of the four items of social stress was 0.62.

### Covariates

Based on previous literature [28], age and sex were selected a priori as covariates. Additionally, psychopathology (psychotic symptoms measured with ESM data) and cannabis use were included as covariates, as psychopathology and cannabis use are associated with smoking and NA and/or stress [20, 29–32]. Psychotic symptoms were assessed as the mean of eight ESM items: “My thoughts are difficult to express,” “My thoughts are influenced by others,” “I cannot get these thoughts out of my head,” “I feel unreal,” “I feel suspicious,” “I hear voices,” “I see things that aren’*t* really there,” and “I’m afraid I’ll lose control” [33–35]. Cronbach’s alpha for these eight items was 0.86.

### Statistical analysis

All analyses were carried out in SPSS Statistics, version 28. Descriptive statistics were used for sample characteristics. Across-time analyses included both smokers and non-smokers, whereas within-subject analyses were restricted to smokers.

ESM data constitute a multilevel structure as multiple observations (level 1) are clustered within participants (level 2). To investigate an association between smoking status and reported daily NA and experienced stress during a six-day period (across assessment times), generalized linear mixed (GLM) models were implemented with a random intercept and an unstructured covariance matrix. Smokers and non-smokers were compared on relevant outcome measures within the three groups (patients, siblings, and controls) separately. Fixed effects were entered sequentially (age and sex, followed by psychotic symptoms and cannabis use). In all analyses with smoking status as an independent variable, non-smokers were the reference category.

Within-subject analyses included only smokers in all three study groups. To investigate the change in daily NA and momentary stress following the event of smoking, new variables were created [19]. A new variable referred to the event of smoking, positively defined if the participant had not smoked on the measurement before (*t*_−1_) and now reported to have smoked a cigarette (*t*_0_). For cases who smoked several times a day, multiple events were taken into account, under the condition of at least two non-smoking measurements before the second series of the day started (i.e. around 180 minutes of cessation). Next, the variable *time_since* was created, which indicated NA and reported stress at measurements before, right after and at the next measurement following the event of smoking approximately 90 minutes later (i.e. NA/stress at *t*_−1_, t_0_ and *t*_+1_). Multilevel analyses were carried out with repeated measures in individual assessments, nested within days, which were nested within individuals. GLM models were conducted with person mean centered values of NA and the stress variables as the dependent variable and *time_since* as the independent variable. In a subsequent step, psychotic symptoms and cannabis use were set as covariates.

### Sensitivity analyses

To account for a possible effect of subsequent smoking reported *t*_+1_, sensitivity analyses were performed. Subsequent smoking at *t*_+1_ was added to the GLM model as a fixed effect.

## Results

### Baseline sample characteristics

Of 486 ESM participants (194 patients, 169 relatives, 123 controls), 411 participants met the ≥ 20 valid measurements criterion (158 patients, 136 relatives, and 117 controls, i.e. 85% of 486). Participants completed on average 41.5 to 48.0 prompts. Mean scores of sample characteristics for these participants are listed in Table 1.

**Table 1.** Sample characteristics for across assessment analyses

	Patients (*n* = 158)	Siblings (*n* = 136)	Controls (*n* = 117)
Smokers	78 (49.4%)	28 (20.6%)	16 (13.7%)
Age, M (SD)	34.1 (8.1)	35.6 (8.8)	40.7 (11.5)
Sex			
Male	106 (67%)	52 (38%)	36 (31%)
Level of education			
Secondary school	22 (14%)	10 (7%)	5 (4%)
High school	25 (16%)	3 (2%)	15 (13%)
Vocational school	80 (51%)	77 (57%)	64 (55%)
University	31 (20%)	45 (33%)	33 (28%)
Ethnicity			
White	141 (89%)	123 (90%)	113 (97%)
Other	17 (11%)	13 (10%)	4 (3%)
Marital status			
Married/living together	32 (20%)	89 (65%)	66 (67%)
Not married/divorced/widowhood	126 (80%)	47 (45%)	33 (33%)
CAPE			
Positive symptoms	0.49 (0.50), *n* = 158	0.08 (0.11), *n* = 135	0.07 (0.10), *n* = 113
Negative symptoms	0.93 (0.56), *n* = 158	0.46 (0.34), *n* = 135	0.43 (0.33), *n* = 113
Depressive symptoms	0.97 (0.60), *n* = 158	0.49 (0.36), *n* = 135	0.51 (0.44), *n* = 113
PANSS, M (SD)			
Positive symptoms	12.2 (7.0)		
Negative symptoms	11.1 (5.0)		
Emotional distress	13.1 (4.9)		

Abbreviations: CAPE, Community Assessment of Psychic Experience, frequency subscales; PANSS, Positive and Negative Syndrome Scale.

### Associations across time

The estimates of the models with smoking status as an independent variable are shown in Supplementary Tables 1 and 2. One significant association was found in patients between smoking status and NA, in a positive direction (*B* = 0.26, SE = 0.12, *p* = 0.036). After adding the extra covariates, cannabis use and psychotic symptoms, significance was lost (*B* = 0.12, SE = 0.09, *p* = 0.223). In siblings and controls, no significant associations were found between smoking status and NA. In none of the study groups, associations were found between smoking status and stress outcome measures.

### Moment-to-moment within-subject analyses

**Figure 1.**
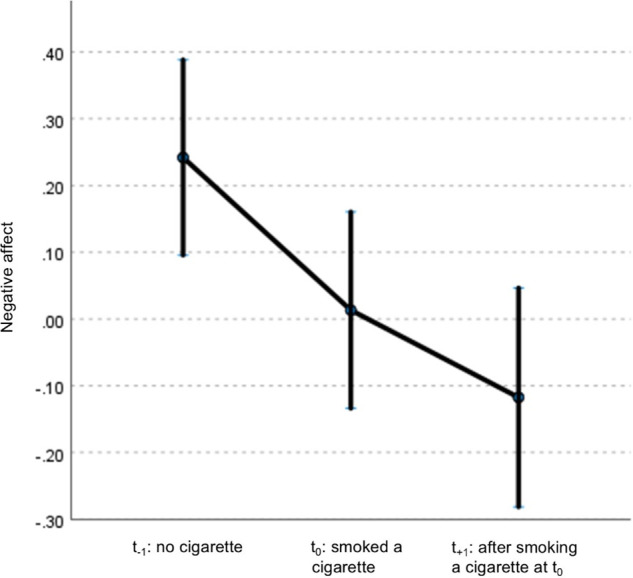
Displays mean centered scores of NA and its 95% confidence interval at different times of assessment: prior to smoking (*t*_−1_), concurrent with smoking (*t*_0_), and the subsequent assessment after smoking (*t*_+1_). Estimates are adjusted for psychotic symptoms and cannabis use.

After excluding non-smokers, analyses included 57 patients (83 events of smoking in total), 29 siblings (54 events), and 20 controls (38 events). General characteristics are listed in Table 2. In patients, NA decreased at *t*_0_ and *t*_+1_ compared to *t*_−1_ and remained significant after adjustment for covariates (Supplementary Table 3; Table 3; Figure 1). Siblings showed a similar pattern in NA, with only t_0_ remaining significant after adjustment (Supplementary Table 4; Table 4). No associations were observed in controls (Supplementary Tables 5 and 6). When accounting for subsequent smoking at *t*_+1_ in the GLM model, the observed time-lagged effect in NA in patients was no longer significant (*t*_+1_: B = −0.23, *p* = 0.084).

**Table 2.** Sample characteristics for moment-to-moment within-subject analyses (smokers only)

	Patients (*n* = 57)	Siblings (*n* = 29)	Controls (*n* = 20)
Age, M (SD)	33.1 (7.5)	33.9 (8.4)	41.8 (11.4)
Sex			
Male	44 (77%)	10 (35%	10 (50%)
Level of education			
Secondary school	10 (18%)	4 (14%)	0 (0%)
High school	11 (19%)	0 (0%)	4 (20%)
Vocational school	28 (49%)	15 (52%)	11 (55%)
University	8 (14%)	9 (31%)	5 (25%)
Ethnicity			
White	49 (86%)	26 (90%)	20 (100%)
Other	8 (14%)	3 (10%)	0 (0%)
Marital status			
Married/living together	8 (14%)	13 (45%)	7 (44%)
Not married/divorced/widowhood	49 (86%)	16 (55%)	9 (56%)
CAPE			
Positive symptoms	0.55 (0.57), *n* = 57	0.06 (0.09), *n* = 28	0.08 (0.20), *n* = 16
Negative symptoms	1.00 (0.62), *n* = 57	0.53 (0.37), *n* = 28	0.46 (0.34), *n* = 16
Depressive symptoms	01.02 (0.65), *n* = 57	0.62 (0.40), *n* = 28	0.54 (0.40), *n* = 16
PANSS, M (SD)			
Positive symptoms	13.1 (7.1)		
Negative symptoms	11.2 (5.1)		
Emotional distress	14.5 (5.8)		

Abbreviations: CAPE, Community Assessment of Psychic Experience, frequency subscales; PANSS, Positive and Negative Syndrome Scale.

**Table 3.** Results of generalized linear mixed model analyses in patients, examining the effect of smoking (*t*_0_) on NA and stress outcomes on *t*_0_ and *t*_+1_ compared with the previous non-smoking assessment (*t*_−1_)

	Patients							
	Negative affect		Event stress		Activity stress		Social stress	
	Estimate (SE)	*p*	Estimate (SE)	*p*	Estimate (SE)	*p*	Estimate (SE)	*p*
*t*_0_	−0.20 (0.08)	**0.015**	−0.12 (0.17)	0.462	−0.13 (0.12)	0.281	−0.24 (0.17)	0.162
*t*_+1_	−0.29 (0.09)	**0.002**	−0.34 (0.17)	0.053	−0.12 (0.13)	0.355	−0.21 (0.18)	0.254

*Note*: Models were adjusted for psychotic symptoms and cannabis use. Values represent estimates (*B*), standard errors (SE) and *p*-values. Bold values indicate statistically significant results (*p* < 0.05).

**Table 4.** Results of generalized linear mixed model analyses in siblings, examining the effect of smoking (*t*_0_) on NA and stress outcomes on *t*_0_ and *t*_+1_ compared with the previous non-smoking assessment (*t*_−1_)

	Siblings							
	Negative affect		Event stress		Activity stress		Social stress	
	Estimate (SE)	*p*	Estimate (SE)	*p*	Estimate (SE)	*p*	Estimate (SE)	*p*
*t*_0_	−0.14 (0.06)	**0.018**	−0.08 (0.18)	0.650	0.09 (0.13)	0.495	−0.06 (0.17)	0.719
*t*_+1_	−0.13 [0.07)	0.077	−0.53 (0.22)	**0.016**	0.10 (0.15)	0.505	−0.14 (0.19)	0.473

*Note*: Models were adjusted for psychotic symptoms and cannabis use. Values represent estimates (*B*), standard errors (SE) and *p*-values. Bold values indicate statistically significant results (*p* < 0.05).

## Discussion

The current study aimed to investigate the daily life associations between smoking, NA, and experienced stress in patients with psychosis, their unaffected siblings, and healthy controls. Across assessments, smoking status was initially associated with higher NA in patients, but this effect disappeared after adjustment for psychotic symptoms and cannabis use. Within-subject analyses showed a short-term decrease in NA after smoking a cigarette in both patients and siblings, which remained after controlling for confounders.

Patients who smoked reported higher NA, although the estimates were small and therefore of modest clinical relevance. This aligns with evidence in the general population [4, 16]. One possible explanation for higher levels of NA in patients who smoke could be coping styles, where patients possibly use smoking as a strategy to cope with experienced negative emotions [10]. However, after controlling for symptoms and cannabis use, smoking status itself was not associated with NA in any group. Instead, mainly psychotic symptoms were associated with increased NA, which is in line with the previously found associations in the ESM study of Klippel et al. [3].

Moment-to-moment analyses revealed that, even after controlling for psychotic symptoms and cannabis use, NA significantly decreased after smoking a cigarette in patients and siblings. However, no sustained effects approximately 90 minutes later were detected when accounting for subsequent smoking. Two mechanisms may explain this pattern. First, the stress-coping model proposes that smoking helps to regain emotional stability in stressful situations [36]. Second, the stress induction model suggests that nicotine deprivation leads to withdrawal symptoms, such as NA and stress, which are alleviated upon smoking [37]. The latter was further supported by the results of our sensitivity analyses in patients, where the time-lagged effect measured at *t*_+1_ of smoking at t_0_ disappeared when controlling for subsequent smoking at *t*_+1_. This could indicate that people smoke a next cigarette before withdrawal symptoms occur. Interestingly, associations were not found in healthy controls, which may be explained by shared vulnerability between patients and their siblings, and not in healthy controls [38]. Furthermore, patients smoke more cigarettes per day than controls, which may produce stronger withdrawal symptoms and thus greater NA relief after smoking [12]. Limited power in the control group may also have contributed. Importantly, no effects were observed for stress outcomes in either across time and within-subject analyses, despite patients’ self-reported motives [6], which challenges the self-medication hypothesis [7].

The main strengths of the current study are the availability of ESM data and the presence of three different study groups with different levels of susceptibility to psychosis. Furthermore, we were able to do both across-assessment and within-subject analyses to investigate cross-sectional associations as well as possible causal relationships. However, several limitations must be noted. First, ESM data contain moment-to-moment data, with high ecological validity. However, this data was based on a subjective self-report of mood experiences, and in this way of collecting data, psychological and biological measures were not taken into account, which could have influenced stress measures. For example, future studies could include between-subject psychological factors such as coping style, and/or biological measures such as nicotine metabolism and cortisol reactivity to further elucidate mechanisms linking smoking, NA, and stress in psychosis. Furthermore, reliance on self-reported data may introduce bias, such as social desirability bias, as participants may not accurately report their smoking behavior and/or (relation with their) emotional states. Nonetheless, ESM has been validated as a reliable method in patients with psychosis and relatives [39]. Second, a response rate cutoff of 20 valid measurements was applied, which may have introduced selection bias. Vermeulen et al. [12] analyzed smoking status in a larger GROUP study cohort and found higher smoking rates than in the current study in all study groups. Furthermore, time series without a non-smoking measurement at *t*_−1_ (e.g. smoking at the first measurement of the day) were excluded, leaving out participants with severe smoking addiction (33 participants in total, i.e. 24%). Compared to the large GROUP cohort [12], the ESM participants were older and showed lower scores on the CAPE questionnaire, which suggests that our cohort represents a better-functioning group. Additionally, patients who participated in the GROUP study [12] represented a relatively high-functioning cohort with lower levels of symptoms compared to the average patient with psychosis. Furthermore, by only analyzing smokers in the within-subject analyses, sample size was limited, leading to potential type 2 errors in controls. These limitations have a potential impact on the generalizability of the findings. Third, in the within-subject analyses, the decision was made to investigate one time moment after a participant smoked a cigarette, to reduce the effect of possible confounders. Furthermore, Berkman et al. reported that mood predicted smoking, mediated by craving, only for 2 hours and not after 4 hours [40]. This implies that the association between mood and smoking is short in duration. However, for future studies, the course of smoking, stress, and NA during a day would be an interesting topic as an addition to the current study and in light of the study of Vaessen et al., who studied the time of recovery of NA during a day in patients with psychosis [19]. Fourth, despite the finding of significant associations, clinical relevance may be negligible because of small estimates. Furthermore, given the explorative design of the current study, statistical significance was set at *p* < 0.05 instead of using a more conservative approach as the Bonferroni correction for multiple testing. Therefore, we introduced the risk of a Type I error. Future studies need to elucidate the clinical relevance of these associations. In the current study, we investigated temporal associations and the potential role of smoking on NA and stress. However, we acknowledge that reverse causation may be at play and should be investigated in future research, for instance by marking stressful moments during the day and subsequently tracking one’s smoking behavior. Furthermore, the cumulative effect of smoking on NA and stress throughout the day warrants further investigation.

In conclusion, no differences in NA were found between smokers and non-smokers when controlling for psychotic symptoms. In the short term, NA decreases after smoking in patients with psychosis and siblings. These findings add to the existing literature by clarifying the complex interplay between smoking and affective states in psychosis and questioning the self-medication hypothesis. Rather than providing lasting stress relief, smoking may temporarily alleviate nicotine withdrawal symptoms, thereby maintaining smoking behavior. From a clinical perspective, these results suggest that smoking cessation for these people is even more complex and clinical attention during smoking cessation treatment on psychoeducation about nicotine withdrawal might be essential. Smoking cessation support in patients with psychosis should explicitly address NA regulation and interventions could benefit from integrating behavioral strategies to manage stress and NA, combined with pharmacological aids to reduce withdrawal symptoms. Future research could investigate the potential supportive effect of targeted interventions using EMI (ecological momentary interventions) as a just-in-time daily life support to manage fluctuations in affect during cessation attempts.

## Supporting information

10.1192/j.eurpsy.2025.10148.sm001 van der Velde et al. supplementary material van der Velde et al. supplementary material

## Data Availability

The data that support the findings of this study are available from the GROUP cohort principal investigators on reasonable request. The data are not publicly available due to containing information that could compromise research participant privacy or consent.

## Abbreviations

CAPE: Community Assessment of Psychic Experience
DSM-IV: Diagnostic and Statistical Manual of Mental Disorders, Fourth Edition
EMI: Ecological Momentary Interventions
ESM: Experience Sampling Method
GLM: Generalized Linear Mixed
GROUP: Genetic Risk and Outcome of Psychosis
NA: negative affect
PANSS: Positive and Negative Syndrome Scale

## Supplementary material

The supplementary material for this article can be found at http://doi.org/10.1192/j.eurpsy.2025.10148.
