# Supplementary material for: Exploring the interplay among smoking, stress, and negative affect in patients with psychosis: An experience sampling method study
Source: Eur Psychiatry. 2025 Dec 29;69(1):e3. doi: 10.1192/j.eurpsy.2025.10148 (PMC12816924; doi:10.1192/j.eurpsy.2025.10148)
Supplement: van der Velde et al. supplementary material [file S092493382510148Xsup001.docx]

|  | Patients | | Siblings | | Controls | |
| --- | --- | --- | --- | --- | --- | --- |
| Outcome variable | Estimate (SE) | p | Estimate (SE) | p | Estimate (SE) | p |
| Negative affect | 0.26 (0.12) | **0.036** | 0.17 (0.13) | 0.186 | 0.08 (0.09) | 0.406 |
| Event stress | 0.04 (0.13) | 0.754 | -0.12 (0.13) | 0.381 | 0.20 (0.12) | 0.106 |
| Activity stress | 0.16 (0.12) | 0.211 | -0.05 (0.13) | 0.717 | 0.13 (0.10) | 0.226 |
| Social stress | 0.04 (0.15) | 0.807 | 0.03 (0.15) | 0.823 | 0.08 (0.12) | 0.520 |
| **Supplementary Table 1**: Results of the generalized linear mixed model analyses with smoking status as independent variable and NA and the stress outcomes as dependent variables across ESM assessments, corrected for age and sex. | | | | | | |

|  | Patients | | Siblings | | Controls | |  |
| --- | --- | --- | --- | --- | --- | --- | --- |
| Outcome variable | Estimate (SE) | p | Estimate (SE) | p | Estimate (SE) | p |  |
| Negative affect | 0.12 (0.09) | 0.223 | 0.11 (0.09) | 0.235 | 0.11 (0.07) | 0.137 |  |
| Event stress | 0.01 (0.13) | 0.969 | -0.13 (0.13) | 0.302 | 0.23 (0.11) | 0.056 |  |
| Activity stress | 0.09 (0.11) | 0.426 | -0.08 (0.12) | 0.510 | 0.14 (0.10) | 0.149 |  |
| Social stress | -0.03 (0.14) | 0.847 | -0.01 (0.13) | 0.960 | 0.09 (0.12) | 0.460 |  |
| **Supplementary Table 2**: Results of the generalized linear mixed model analyses with smoking status as independent variable and NA and the stress outcomes as dependent variables across ESM assessments, corrected for age, sex, psychotic symptoms and cannabis use. | | | | | | | |

|  | Patients | | | | | | | |
| --- | --- | --- | --- | --- | --- | --- | --- | --- |
|  | NA | | Event stress | | Activity stress | | Social stress | |
|  | Estimate (SE) | p | Estimate (SE) | p | Estimate (SE) | p | Estimate (SE) | P |
| t_0_ | -0.22 (0.09) | **0.016** | -0.12 (0.17) | 0.478 | -0.15 (0.13) | 0.238 | -0.25 (0.17) | 0.141 |
| t_+1_ | -0.36 (0.11) | **0.001** | -0.37 (0.18) | **0.039** | -0.16 (0.14) | 0.250 | -0.23 (0.18) | 0.197 |
| **Supplementary Table 3**: Results of generalized linear mixed model analyses in patients, examining the effect of smoking (t_0_) on NA and stress outcomes on t_0_ and t_+1_ compared with the previous non-smoking assessment (t_-1_). Values represent estimates (B), standard errors (SE) and p-values. | | | | | | | | |

|  | Siblings | | | | | | | |
| --- | --- | --- | --- | --- | --- | --- | --- | --- |
|  | NA | | Event stress | | Activity stress | | Social stress | |
|  | Estimate (SE) | p | Estimate (SE) | p | Estimate (SE) | p | Estimate (SE) | p |
| t_0_ | -0.22 (0.08) | **0.009** | -0.09 (0.18) | 0.608 | 0.07 (0.13) | 0.622 | -0.07 (0.17) | 0.661 |
| t_+1_ | -0.22 (0.10) | **0.030** | -0.54 (0.22) | **0.013** | 0.07 (0.15) | 0.647 | -0.15 (0.19) | 0.435 |
| **Supplementary Table 4**: Results of generalized linear mixed model analyses in siblings, examining the effect of smoking (t_0_) on NA and stress outcomes on t_0_ and t_+1_ compared with the previous non-smoking assessment (t_-1_). Values represent estimates (B), standard errors (SE) and p-values. | | | | | | | | |

|  | | | | | | | | |
| --- | --- | --- | --- | --- | --- | --- | --- | --- |
|  | Controls | | | | | | | |
|  | NA | | Event stress | | Activity stress | | Social stress | |
|  | Estimate (SE) | p | Estimate (SE) | p | Estimate (SE) | p | Estimate (SE) | p |
| t_0_ | -0.06 (0.09) | 0.467 | -0.16 (0.24) | 0.510 | 0.05 (0.16) | 0.747 | 0.29 (0.29) | 0.326 |
| t_+1_ | 0.03 (0.09) | 0.731 | 0.04 (0.24) | 0.862 | 0.29 (0.17) | 0.097 | 0.05 (0.31) | 0.878 |
| **Supplementary Table 5**: Results of generalized linear mixed model analyses in controls, examining the effect of smoking (t_0_) on NA and stress outcomes on t_0_ and t_+1_ compared with the previous non-smoking assessment (t_-1_). Values represent estimates (B), standard errors (SE) and p-values. | | | | | | | | |

|  | Controls | | | | | | | |
| --- | --- | --- | --- | --- | --- | --- | --- | --- |
|  | NA | | Event stress | | Activity stress | | Social stress | |
|  | Estimate (SE) | p | Estimate (SE) | p | Estimate (SE) | p | Estimate (SE) | p |
| t_0_ | -0.04 (0.09) | 0.690 | -0.12 (0.24) | 0.605 | 0.11 (0.15) | 0.476 | 0.33 (0.29) | 0.262 |
| t_+1_ | 0.07 (0.10) | 0.470 | 0.04 (0.24) | 0.867 | 0.30 (0.17) | 0.077 | 0.08 (0.31) | 0.807 |
| **Supplementary Table 6:** Results of generalized linear mixed model analyses in controls, examining the effect of smoking (t_0_) on NA and stress outcomes on t_0_ and t_+1_ compared with the previous non-smoking assessment (t_-1_). Models were adjusted for psychotic symptoms and cannabis use. Values represent estimates (B), standard errors (SE) and p-values. | | | | | | | | |
